# The immediate and lasting balance outcomes of clinical falls-prevention programs: A non-randomised study

**DOI:** 10.1371/journal.pone.0299146

**Published:** 2024-03-14

**Authors:** Candice K. Oberholster, Carolyn J. Taylor, Minh Huynh, Brett A. Gordon

**Affiliations:** 1 Holsworth Research Initiative, La Trobe Rural Health School, La Trobe University, Bendigo, Victoria, Australia; 2 The Royal Melbourne Hospital, Allied Health (Physiotherapy and Exercise Physiology), Melbourne, Victoria, Australia; 3 La Trobe Rural Health School, La Trobe University, Bendigo, Victoria, Australia; 4 La Trobe School of Allied Health, Human Services and Sport, La Trobe University, Melbourne, Victoria, Australia; Iran University of Medical Sciences, ISLAMIC REPUBLIC OF IRAN

## Abstract

**Purpose:**

Exercise-based falls-prevention programs are cost-effective population-based approaches to reduce the risk of falling for older adults. The aim was to evaluate the short-term and long-term efficacy of three existing falls-prevention programs.

**Methods:**

A non-randomized study design was used to compare the immediate-post and long-term physical outcome measures for three falls prevention programs; one high-level land-based program, one low-level land-based program and a water-based Ai Chi program. Timed-up-and-go (TUG), five-times sit-to-stand (5xSTS), six-minute walk test (6MWT) and six-meter walk test were assessed at baseline, post-program, and at six-months follow-up. Linear mixed models were used to analyze between- and within- group differences, with the high-level land-based program used as the comparator.

**Results:**

Thirty-two participants completed post-program assessment and 26 returned for follow-up. There was a difference in the age (years) of participants between programs (*p* = 0.049). The intercept for TUG and six-meter walk test time was 47.70% (23.37, 76.83) and 32.31s (10.52, 58.41), slower for the low-level group and 40.49% (17.35, 69.89) and 36.34s (12.75, 64.87), slower for the Ai Chi group (*p* < 0.01), compared with the high-level group. Mean time taken to complete the TUG was less both immediately post-program and at 6-month follow-up (*p* = 0.05). Walking speed for the six-meter walk test was only faster at six-months (p < 0.05). The 5xSTS duration was significantly reduced only at post-intervention (p < 0.05).

**Conclusion:**

These results indicate land-based and water-based falls-prevention programs improve physical outcome measures associated with falls-risk and many improvements are maintained for six months after the completion of the program. (Retrospective trial registration: ACTRN1262300119069)

## Introduction

In an aging society, where people are living longer and have more chronic disease, falls are an increasing problem [1]. In Australia, one third of adults over 65 years of age will have at least one fall per year, and adults with neurological conditions are three times as likely to fall compared with their age matched counterparts [2]. Poor balance and fear of falling can lead to reduced activity participation, deconditioning and subsequent frailty, which creates a perpetual cycle of decline, reduced quality of life and risk of falls [3–5]. People who fall often restrict their activity due to previous fall-related physical and psychological injury, and this often results in a reduction in walking confidence and functional decline [6, 7].

Exercise-based falls-prevention programs have demonstrated a reduced risk of falls [8], and a group delivery format can provide a cost-effective population-based approach [9–13]. Exercise can reduce the number of falls and the severity of fall-related injuries by addressing many of the associated modifiable and measurable risk factors, such as muscle strength, static and dynamic balance, walking endurance, gait speed, step length and confidence [6, 8, 14]. For optimal effectiveness, exercise should be completed regularly and focus on multiple functional capacities including coordination, agility, balance, mobility, muscle strength and flexibility, and include multi-sensory training such as walking, stair climbing, and carrying objects [7, 10, 13, 15]. The greatest reduction in falls risk has been observed in programs that include challenging balance tasks completed for more than three hours per week [10, 12, 13] and where at least 30% of the program comprised of balance training [13]. Some studies have demonstrated multi-function exercise interventions, like those that include a combination of balance, strength, and coordination exercises, can improve quality of life, cognitive function, protective reflex speed and, in the event of a fall, the capacity of soft tissues to absorb energy, thereby diminishing the impact force and injury potential of falls [5, 7, 15, 16].

There is also growing body of evidence that supports the use of water-based exercise for improving balance, reducing risk of falls, and fear of falling in older adults and individuals with neurological conditions [17–19]. Water-based therapy has been reported to be more effective than land-based exercise in certain neurological conditions, such as Parkinson’s disease and multiple sclerosis [19, 20]. Water-based exercise utilizes the inherent properties of the water, including buoyancy, viscosity, turbulence, and hydrostatic pressure, to challenge and improve balance [17]. Ai Chi is a water-based exercise program based upon Tai Chi and was developed by Jun Konno in 1993 [21] and has been reported as effective for improving balance in older and neurological populations [17, 22].

Short-term, immediate post-intervention benefits of exercise for reducing falls risk have been established through clinical trials [10]. However, the long-term effects of these programs, once the interventions are completed, are not well understood in clinical practice [23]. The few studies to report follow-up of falls-prevention programs have not presented reassuring data, with some reporting participants returning to pre-program falls risk levels within 3-months [24, 25]. A systematic review by Sherrington and colleagues [10] concluded that ongoing exercise is required to maintain gained benefits, as the ability for participants to maintain improved strength and balance outcomes once programs have ceased is poor. Critically, this evidence was derived from research clinical trials that inform how falls-prevention programs are delivered clinically, but the effect of in-practice real world falls-preventions programs are yet to be systematically investigated. Furthermore, it is unknown if post-program decline occurs at the same rate across different falls-prevention programs following in-practice clinical programs.

Therefore, the primary aims of this study was to evaluate the effectiveness of recommendations from previous trials and their translation to clinical practice. This was achieved through the evaluation and comparison of the short-term efficacy of two land-based and one water-based falls-prevention programs on physical outcomes associated with falls risk. The secondary aims were to assess if any change in physical outcomes were maintained at six-months follow-up and if the type of falls-prevention program had an influence on follow-up outcomes.

## Materials and methods

### Study design

A non-randomized study design was used to evaluate outpatient exercise-based falls-prevention programs at a major Australian metropolitan hospital. Three existing falls-prevention programs were evaluated for efficacy to improve participant physical function outcomes associated with falls risk. Assessments of physical function were conducted at baseline, immediately after the 10-week falls-prevention programs, and at six-month follow-up.

This study was approved by the Melbourne Health and La Trobe University Human Research Ethics Committees (LNR/16/MH/211). The original approved inclusion criteria were modified initially participants were required to complete the 5xSTS in under 15 seconds, however, this criteria was amended to allow participants to complete the 5xSTS without any time restriction. This was done to allow a more representative demographic sample of individuals referred to the falls-prevention programs. This amendment was approved by both ethics committees. Participants were recruited between 26^th^ of April 2017 and 3^rd^ September 2018. All participants provided verbal and written consent after receiving verbal and written explanation of the study protocol, and potential risks and benefits. The protocol was retrospectively registered with the ANZCTR (ACTRN12623001190695) and the reporting conforms with the TREND statement (S1). At the time of completion, ethical approval did not require clinical registration and the authors did not consider the protocol to be a clinical trial requiring registration.

### Setting and participants

The hospital was a major tertiary metropolitan hospital which offered three outpatient falls-prevention programs, each facilitated by an accredited exercise physiologist and an allied health assistant. All participants who were referred to one of the three falls-prevention exercise programs were considered for recruitment. Referral process and program intervention were completed following standard clinical practice for the hospital; study participants received the same care as other patients in the programs. Program selection was based on the clinical judgement of the referring clinician, rather than randomization, according to participant physical function and preference, as per standard clinical care. Requirements for referral to the outpatient falls-prevention programs included: (i) score in the moderate or high risk category on Falls Risk for Older People–Community Setting (FROP-COM) [26] or a history of falling and/or other indicators for risk of falls, such as reduced lower limb strength, reduced static and dynamic balance, and fear of falling; (ii) ability to perform a dynamic step test [27] without upper limb support; (iii) ability to perform a five times sit-to-stand [28]; (iv) completion of all relevant risk screening to participate in referred protocol (for example the hydrotherapy risk screen if referred to the water-based programs). The principal researcher and assessors had no influence on which participants were referred and/or to which intervention.

Potential participants were excluded from the study if they were unable to provide informed consent due to cognition, a language barrier (where use of interpreter was declined), or where assessors were unable to complete initial assessment prior to the patient commencing the program due to time and staffing limitations. Participants were able to complete complementary therapy alongside the intervention, as per usual care.

### Sample size

Sample size was calculated using the estimates of effect size (d = 0.50) for interventions comparing moderate- and high-challenge balance exercises published in a review by Sherrington et al.[29], and pre- and post-intervention TUG test data comparing high- and low-intensity exercise in participants suffering stroke (d = 0.29) [30]. An a-priori power analysis for a repeated-measures analysis of variance with three groups and three repeat measures (pre, post and follow-up), with an alpha value of 0.05 for significance and a beta value of 0.80 was calculated using G*Power software (3.1.9.2 for Windows). Sample size estimates ranged from nine participants (d = 0.50; providing statistical power of 86%) to 27 participants (d = 0.29; providing statistical power of 82%). Therefore, the intention was to recruit at least 30 participants, with a minimum of 10 participants in each program, to provide sufficient power to determine change in the primary outcome (TUG). Where necessary, participants were replaced through additional recruitment if they withdrew or failed to complete the intervention.

### Procedures

When participants were referred to one of the three falls-prevention programs and consented to the study, they completed physical outcome assessments at baseline then completed a 10-week falls-prevention program. At discharge, the physical outcome assessments were repeated, and participants were educated in line with standard practice to continue to remain active. Where appropriate, participants were provided with a home exercise program, or information on suitable community exercise options by their referring therapist. Participants were contacted to return for follow-up physical outcome assessments six months after discharge.

### Exercise protocols

The two land-based falls-prevention programs were high-level balance (HLB) and low-level balance (LLB), and both programs used balance and strength-based exercises. These programs were developed based on recommendations from previous clinical trials [13, 30] and in accordance with available hospital resources. Programs were conducted in the hospital rehabilitation gym. The land-based falls-prevention programs differed only in exercise intensity/difficulty and volume, with the LLB program prescribed at a lower intensity and progressed more gradually than the HLB program. Exercises for both programs were prescribed and progressed based on participant ability and were individualized by adjusting the sets and/or repetitions, changing base of support (wide stance verses tandem stance) and included visual instability, multitasking or cognitive components. Each exercise session was 45–50 minutes and conducted twice a week. As there is no standard measure to prescribe balance intensity [13], participants were progressed as the supervising therapist judged to be challenging but with-out compromising safety. Due to the complexity of the body’s balance systems, this was completed in numerous ways, including but not limited to, the shifting of center of mass, reducing base of support, reducing upper limb support, reducing vision/gaze stability and the addition of cognitive task while completing exercises [30] (see Table 1 for example exercise). Participants undertook exercises within parallel rails with chairs positioned at each end. Whilst the rails were available for safety purposes, participants were encouraged not to use rails unless necessary and close therapist supervision was used when participants completed particularly challenging balance exercises (i.e. standing on dura disc with eyes closed). A maximum of six participants participated in each exercise group session, supervised by an accredited exercise physiologist who was assisted by an allied health assistant to ensure staff: participant ratios were not more than 1:3.

**Table 1:** Example exercises.

Land-based: HLB and LLB	Water-based: Ai Chi*
Squats with or without use of a chair^#^ Heel/toe-raises (balance focus)^#^ Step-ups^#^ Step-downs^#^ Walking in rails (forwards, backwards, tandem) Multi-tasking activities on different surfaces/ base of support sizes (counting, head-turns, throwing) Marching on the spot Foam standing with changing base of support size and/or gaze stability/eyes closed Step-tapping Stepping over obstacles Rocking ant/posterior or laterally on wobbleboards Balancing/walking on Dura Disc Using a stepper machine Single-leg and tandem standing	Contemplating Floating Uplifting Enclosing Folding Soothing Gathering Freeing Sifting Accepting Accepting with Grace Rounding Balancing Half circling Encircling Surrounding Nurturing Flowing Reflecting

HLB = High level balance, LLB = Low level balance

^#^ 1–2 sets of 8–15 repetitions of each exercise were completed.

* Repetitions of 8–15 of each posture were completed before progressing to the next kata.

The water-based falls-prevention program utilized Ai Chi principles and postures conducted in a hydrotherapy pool (Table 1). The pool temperature was 34 degrees Celsius and had a sloped floor. Participants were encouraged to complete postures in chest depth water as per Ai Chi guidelines [22]; however, some commenced the session in the shallow water and gradually progressed to deeper water throughout the session and/or program as confidence and stability improved. Each session consisted of 5-minutes of walking in the water followed by completing a sequence of up to nineteen Ai Chi movements, or katas, for 30–40 minutes and then a 5-minute cool down. Katas move sequentially through gradually narrower bases of support, from bilateral to unilateral movements while also increasing difficulty with gaze instability and more complex motion. All katas were completed in a slow and continuous motion to prevent ‘gripping’ on the water. Movement prescription was adjusted on clinical judgement based on participant ability, with participants progressed to the most challenging kata they were able to complete with-out significant loss of balance. Each exercise session was 45–50 minutes and conducted twice a week. Rails were available along the edge of the pool and participants were oriented to the entry/exit and familiarized with emergency pool procedures prior to commencing the program. A maximum of six participants participated in each exercise group session, supervised by an accredited exercise physiologist who was assisted by an allied health assistant to ensure staff: participant ratios were not more than 1:3, with at least one staff member in the pool with the participants. Both staff completed annual pool-rescue training. An additional pool-rescue trained staff-member was always available nearby.

### Outcomes

Assessments were conducted by clinical staff (accredited exercise physiologist or physiotherapist) or final year exercise physiology student (under supervision). While assessors were not blinded, strict standard protocols and instructions were followed to minimize potential bias. In addition to functional capacity, participant characteristics were recorded at baseline assessment. These included age, sex, sociocultural background/preferred language, primary diagnosis, and comorbid conditions.

The TUG test is a measure of agility and dynamic balance and is a valid and reliable screening tool for functional mobility and risk of falls in community-dwelling older adults and neurological populations [31–33]. Wrisley and Kumar [34], determined that a TUG score of >12.3 seconds was the optimum cut-off score for predicting falls in the following 6-months, in adults aged 60 to 90 years. However, a systematic review by Barry et al., [31] cautioned against using TUG in isolation, as the nature of falls is complex. Participants sat in a 46 cm high chair, stood on the command ‘go’ and walked forward to a marker at three meters before turning around, returning to their chair and sitting back down as quickly and as they safely could. The time (s) taken to complete this task was measured using a hand-held stopwatch and recorded.

The 5xSTS test evaluates functional lower limb strength with excellent validity and good reliability for predicting recurrent falls [35]. Using a 46 cm high chair, the participants were instructed to stand up and sit down as quickly as they could safely five times in a row. The time (s) taken to complete the task was measured using a hand-held stopwatch and recorded.

The six-meter walk test is a significant predictor of recurrent falls with high-moderate reliability [35], and can be used to calculate the participants average step length and walking speed. The participants were instructed to walk as quickly as possible for ten meters across a marked track. The number of steps taken to complete the middle six meters manually counted from when the participants first foot crossed the two-meter mark until it crossed the eight-meter mark. Time taken to walk the middle six meters was also recorded. This data was used to calculate step length and walking speed.

The 6MWT has excellent test-retest reliability to assess walking endurance [36–38]. The participants were instructed to walk as far as possible in six minutes on a 20-meter indoor track. Participants were allowed to rest at any stage during the six minutes, but the time did not stop. Total distance walked was recorded in meters.

The International Physical Activity Questionnaire (IPAQ) is a self-reported measure of an individual’s habitual physical activity which has been validated in adults up to 84 years of age [39], however, it has been reported that those aged over 65 years tend to overestimate moderate-intensity exercise more so than younger aged groups. Participants were asked to complete the questionnaire (where required, the options were read to the participant) and metabolic equivalents (METs) minutes per a week were calculated.

Fear of falling was assessed using the Modified Falls Efficacy (MFES) questionnaire. This questionnaire consists of 14 questions, each relating to a particular task and assesses how confident the participant feels they can complete the task on an 11-point scale of 0 (not confident at all) to 10 (completely confident). The MFES has high internal consistency and high retest reliability (ICC = .93) [40]. The mean score for each participant was recorded and provided as a percentage [40].

### Statistical methods

All statistical analyses were conducted using R (R Core Team, 2020). Demographic and baseline outcome data were analyzed descriptively. At each assessment point there were participant dropouts, which resulted in missing data for the post-intervention and 6-month follow-up assessment points. Due to the resultant small sample size, and the desire to avoid in-filling missing data with estimates, alternate statistical approaches to the repeated measures ANOVA were used. The use of linear mixed models (LMM) allowed comparison of data from groups of unequal distribution and with expected differences in baseline parameters. Linear mixed models are typically seen as an extension of the general linear model and are appropriate for modelling within-participant parameters because they work around the general linear assumption that data points are independent of each other [41]. In a within-participant design, participants provide multiple data points which are correlated with each other because they come from the same individual. Therefore, using a mixed model allows for systematically accounting for both item-level and participant-level variability [41], something that analyses such as repeated measures ANOVA are not suitable for.

Linear mixed models were used to examine if TUG, 6MWT, six-meter walk and 5xSTS differed between programs across the three assessment points. The HLB program was used as the reference group.

The LMM residuals were checked against a normal distribution by visual inspection of a quantile-quantile plot. Dynamic balance, walking speed and step length did not approximate a normal distribution and therefore, were log-transformed. The LMMs included fixed effects program × time and a random intercept for participant ID. The initial LMMs identified no program × time effects for any of the responses, indicating there was insufficient evidence to conclude that indicators of falls risk are influenced differently with any of the three clinical protocols. Clinical diagnosis and sex were not included as covariates within the LMMs as the small numbers would not yield meaningful results. Model fixed effects parameters and random effects variance components were back transformed (for the log-transformed variables) and are reported as percentage differences from the reference levels (pre-test and HLB respectively). These models were refitted to examine the main effects, independent of any interactions. Program × time effects are reported in S1 Table in S1 File (S2 Table in S1 File, S1 Dataset).

The difference in the response variables from baseline to each other time point (post-program and 6-month follow-up) were estimated, and 95% confidence intervals (95% CI) were used to denote the imprecision of fixed effects parameter estimates. Due to the dependent nature of the study design, a repeated measures standardized mean difference (SMDrm) was calculated to represent the size of the effects and interpreted using the following descriptors: trivial (<0.2), small (0.2–0.6), moderate (0.6–1.2), large (1.2–2.0), very large (>2.0) [42]. The SMDrm represents the size of effect an individual might expect to experience with the protocol as opposed to the size of effect that might be expected by a group of individuals with other measures of effect. Unless otherwise indicated, data are presented as mean ± standard deviation (SD) or median and interquartile range (IQR).

## Results

**Fig 1:**
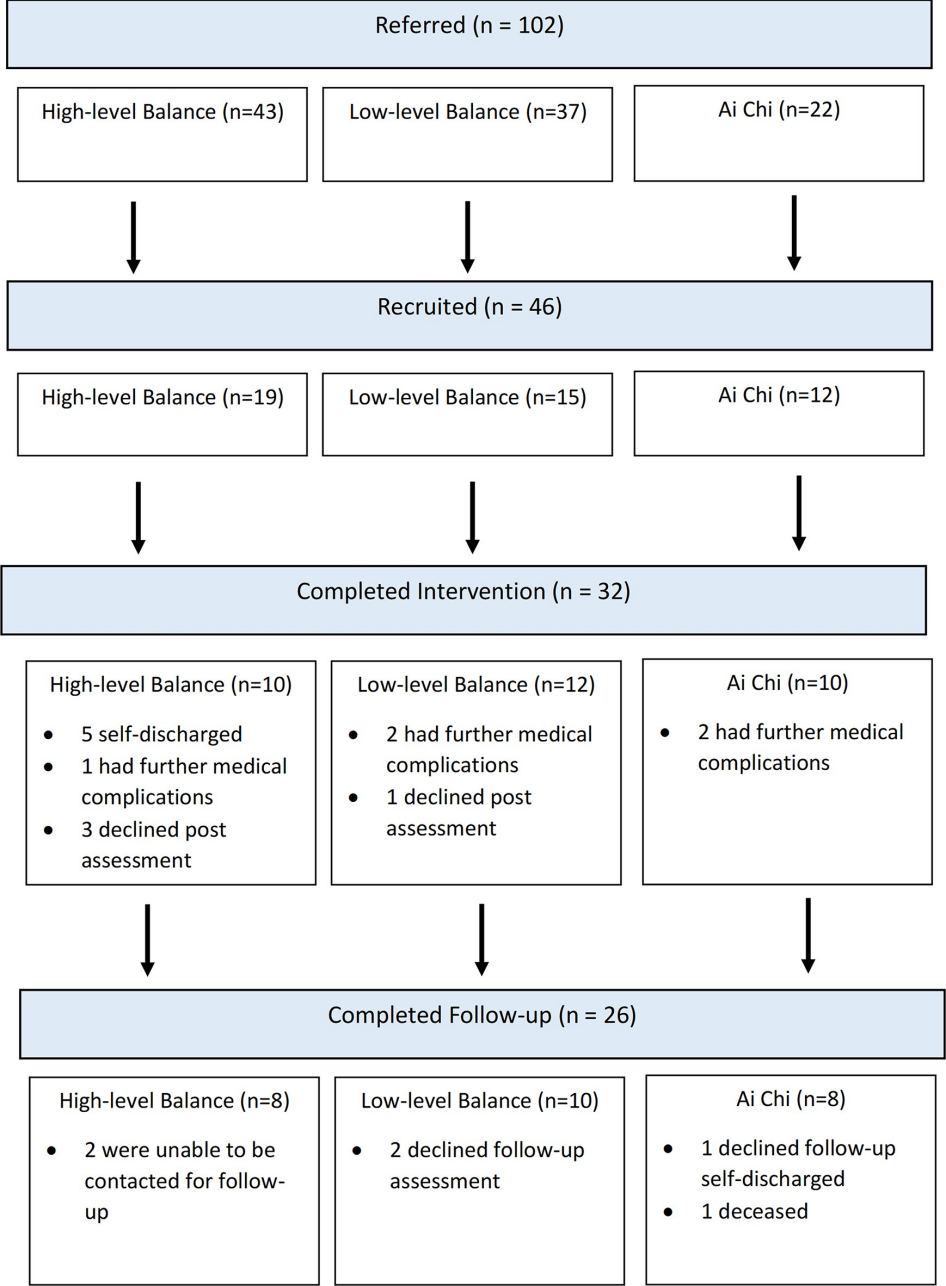
Participant flow diagram.

During the 18-month recruitment period, 102 patients were referred into one of the three falls-prevention programs (Fig 1). Fifty-four percent of patients were referred due to age-related changes, 26% due to neurological conditions, and 19% due to neurological-progressive conditions. Of these, 46 patients consented to participate in the study, 19 in the HLB program, 15 in the LLB program and 12 in the Ai Chi program. Thirty-two (70%) participants completed the post-intervention assessment and 26 (57%) returned for six-month follow-up assessment. Reasons for dropouts included participants self-discharging from the hospital service (n = 5), further medical complications (n = 5), withdrawing from the study (n = 3), participants were unable to be contacted (n = 2), and one (n = 1) participant had died prior to follow-up. There were no reportable adverse events during the program.

Descriptive statistics for demographic and physical activity variables (diagnosis, sex and IPAQ) are reported in Table 2. There was a difference in age (p = 0.049) between the three programs at baseline. As the participants were assessed and referred to their respective programs based on their physical capacity prior to commencing the program, there was an expected significant difference in physical function between programs at baseline. Consequently, participants in the HLB program had higher scores compared with participants in the LLB program in most physical outcome measures (Table 3). There were also significant differences between HLB and Ai Chi programs at baseline for 5xSTS and functional reach (Table 3). There were no significant differences in baseline physical function outcomes between LLB and Ai Chi programs for any measures. All programs had similar baseline scores for self-reported confidence levels (p > 0.05). Mean scores for programs at each assessment point can be found in S2 Table in S1 File.

**Table 2:** Descriptive statistics between groups–characteristics.

	Diagnosis n (%)			Gender n (%)		Age (years)	IPAQ (MET/min/week)
Group	Age-related changes	Neurological	Neurological-progressive	Female	Male	Mean (SD)	Mean (SD)
High level balance	10 (52.6%)	5 (26.3%)	4 (21.1%)	13 (68.4%)	6 (31.6%)	71.5 (10.6)	2510.0 (2339.1)
Low level balance	9 (60.0%)	4 (26.7%)	2 (13.3%)	10 (66.7%)	5 (33.3%)	78.5 (9.5)	1998.7 (2174.1)
Ai Chi	6 (50.0%)	3 (25.0%)	3 (25.0%)	8 (66.7%)	4 (33.3%)	68.6 (11.6)	2553.0 (2017.1)

IPAQ = International Physical Activity Questionnaire, MET = metabolic equivalents

**Table 3:** Descriptive statistics between groups—baseline physical capacity.

Outcome	High Level Balance (n = 19)	Low Level Balance (n = 15)	Ai Chi (n = 12)	Between Group *p*-value
6-min walk (m) *	357 (305–409) ǂ	274 (230–319)	280 (224–337)	0.026
6-meter walk (s) *	5.0 (4.3–5.7) ǂ	7.2 (6.3–8.1)	7.5 (5.0–10.2)	0.01
Timed-up and go (s) *	10.0 (8.5–11.6) ǂ	17.2 (12.2–22.1)	15.4 (12.1–18.7)	0.004
5 x sit to stand (s) *	12.8 (10.4–15.2) ǂ φ	17.7 (15.1–20.0)	19.2 (15.0–23.3)	0.004
6-meter walk (steps) **	10 (8–11) ǂ	12 (10–14)	11 (10–14)	0.017

* Reported in mean and 95% CI

**reported in median and IQR, ǂ significant difference between HLB and LLB, φ significant difference between HLB and Ai Chi, † significant difference between LLB and Ai Chi

There were significant program effects for dynamic balance (measured using TUG test; *F* = 12.66, *p* < 0.001), lower limb strength (measured through 5xSTS; *F* = 7.17, *p* = 0.002), walking speed (*F* = 8.08, *p* = 0.001), step length (*F* = 6.09, *p* = 0.01, and walking endurance (measured through 6MWT; F = 4.26, p = 0.021). Walking endurance and dynamic balance were lower for the LLB program in comparison to the HLB program with large or very large magnitudes of effect (Table 4). Lower limb strength was also lower for the LLB program compared to the HLB program, with a moderate magnitude of effect (Table 4). A similar pattern was observed for the Ai Chi program in comparison to the HLB program, although the magnitudes of effect were not as large for walking performance and dynamic balance, but the magnitude of effect for lower limb strength was larger (Table 4).

**Table 4:** Model fixed effects regression parameters.

	6–minute walk test (m)		6–meter walk test–time (% change)		6–meter walk test- steps (% change)		Timed-up and Go (% change)		Five x sit-to-stand (s)	
Fixed Effects	Mean (95%CI)	SMD_rm_	Mean (95%CI)	SMD_rm_	Mean (95%CI)	SMD_rm_	Mean (95%CI)	SMD_rm_	Mean (95%CI)	SMD_rm_
Intercept	324.34(290.75, 357.94) ^#^		5.70 (5.26, 6.23) ^#^		10.59 (9.97, 11.36) ^#^		11.70 (10.7, 12.68) ^#^		15.45 (14.08, 16.82) ^#^	
LLB ^a^	-87.53(-160.51, -14.54) *	2.31	32.31 (10.52, 58.41) ^^^	1.75	20.92 (5.13, 39.1) *	1.58	47.70(23.37, 76.83) ^#^	2.05	2.88(-0.03, 5.79)	0.89
Ai Chi ^a^	-66.79(-139.17, 5.59)	1.76	36.34 (12.75, 64.87) ^^^	1.94	20.92 (4.08, 40.49) *	1.58	40.49(17.35, 69.89)	1.79	5.78 (2.81, 8.74) ^#^	1.78
Post-Invention ^b^	9.43(-10.54, 29.41)	0.25	-6.76(-13.93, 1.01)	0.44	-3.92(-9.52, 2.02)	0.33	-10.42 (-18.13, -1.98) *	0.58	-1.67(-3.26, -0.08) *	-0.52
6-Month Follow-up ^b^	21.35(-0.24, 42.94)	0.56	-9.52 (-17.3, -1) *	0.63	-4.88(-17.3, -1.0)	0.42	-11.31(-19.75, -1.98) *	0.63	-1.44(-3.18, 0.29)	-0.44
IPAQ	0.01(0.0, 0.01)	0.00	0.00(0.00, 0.00)	0.00	0.00(0.00, 0.00)	0.00	0.00(0.00, 0.00)	0.00	0.00(0.00, 0.00)	0.00
MFES	134.17 (50.84, 217.5) ^^^	3.54	-19.75(-41.73, 10.52)	1.38	-17.30(-35.6, 6.18)	1.58	-18.94(-43.45, 16.18)	1.11	-7.2(-13.27, -1.13) *	-2.22
**Random Effects**										
Between participant SD	95.19		1.25		1.19		1.23		8.60	
Within participant SD	37.95		1.17		1.13		1.21		10.65	
ICC	0.86		0.65		0.67		0.56		0.39	

^a^ Reference = High Level Balance

^b^ Reference = pre, SD = standard deviation, SMD_rm_ = standardised mean difference (repeated measures)

# p < .001, ^^^p < .01

* p < .05, 95%CI = 95% confidence interval

There were significant time effects for the TUG test (*F* = 4.90, *p* = 0.01), 5xSTS (*F* = 4.98, *p* = 0.010), walking speed (*F* = 4.23, *p* = 0.020), and walking endurance (F = 3.79, p = 0.029). There were small magnitudes of effect for walking endurance, dynamic balance, and lower limb strength indicating improvements following the programs from baseline to post-program (Table 4). When baseline outcomes were compared to six-month follow-up outcomes, the magnitude of effect remained in a positive direction and indicated small changes (Table 4).

LMMs for IPAQ demonstrated no significant effect across program nor time. However, for MFES there was a significant time effect (*F* = 4.05, *p* = 0.022), with MFES scores lowest for the baseline and equivalent for the post-program and follow-up tests (Table 4). The program effect for MFES was not statistically significant. When falls efficacy, physical activity, and demographic characteristics were included into the model as covariates (Table 4), only falls efficacy (MFES) had a significant effect on walking endurance (mean estimate = 135.45, 95% CI = 55.17, 215.72, *p* = 0.001) and 5xSTS (mean estimate = -8.16, 95% CI = -14.12, -2.21, *p* = 0.007). While not statistically significant, MFES had a moderate effect (SMD_rm_ = 1.11) on TUG, and large effects on 6-meter walk time (SMD_rm_ = 1.38) and 6-meter walk steps (SMD_rm_ = 1.58). There was no observed effect for physical activity, age, diagnosis, or sex.

## Discussion

This study demonstrated an improvement in physical outcomes associated with falls risk (i.e. dynamic balance, lower limb strength, gait speed and walking endurance), immediately after all falls-prevention programs. Further, participants maintained or improved outcome scores at six-months follow-up, irrespective of program. There was no change for fear of falling.

Small to moderate size improvements were observed in all measures of physical function immediately post- program compared with baseline. The findings of the current study are consistent with previous studies that have demonstrated physical function, as measured by physical outcomes associated with falls risk, improves after participation in an exercise-based intervention [14, 16, 43–47]. A systematic review by Sherrington et al., [48] reported, with a high-certainty of evidence, exercise programs that involve balance and functional exercises, reduce the rate of falls in community dwelling older adults. The same systematic review reported Tai Chi and similar programs, which include multidimensional activities (e.g. resistance exercises, balance and functional exercises), are likely to reduce falls [48]. This has been corroborated by other studies [12, 13, 49–52]. Similarly, an increasing number of studies have demonstrated Ai Chi can improve participant balance and gait, and thereby reduce falls risk [17, 18, 49, 53–55].

An important finding from the current investigation is that all falls-prevention programs were efficacious in improving physical function associated with falls risk. This finding is encouraging as recent recommendations suggest that thrice weekly exercise sessions [10, 12, 13], or an accumulation of 40-hours exercise over the course of a program, is required to significantly reduce falls incidence [13]. In contrast, this study provided only ten weeks, or 20 hours contact, due to resource constraints, yet improvements in physical function were found. The improvements seen in this study, compared to those that provided a similar number of contact hours [56], might be attributed to the higher proportion of complex balance exercises completed in both the land- and Ai Chi programs. As land- and water-based exercise protocols elicited improvements in physical outcomes, independent of factors such as diagnosis and functional status, individual preference and post discharge accessibility to similar activity-types should be considered when determining an appropriate referral pathway to optimize continued exercise participation [57, 58].

There were no differences for change in physical function in response to either land- or water-based falls prevention programs in the current study. This is despite an expected difference in initial physical capacity for participants in most parameters assessed between programs. Ai Chi has previously been reported to be equally as effective or superior for improving balance risk factors in some population sub-groups, such as individuals who have experienced a stroke, Multiple Sclerosis or Parkinson Disease [17–19, 53–55], compared with conventional land- or water-based exercise programs. The allocation of participants into programs based on participant physical function, preference and ongoing accessibility in this study may be a key difference to approaches used in previous studies. Clinically, providing protocols that match an individual’s capacity and preferences could be an important contributor to ongoing exercise behaviors [57–59].

Similar changes in physical function outcomes were found between baseline and post-program, and baseline and 6-month follow-up for all programs. This suggests participants were able to maintain improvements for at least 6-months after discharge. Further, there is some indication that participants continued to improve in dynamic balance and walking speed as indicated by TUG and 6-meter walk test. The ability to maintain improved walking speed after exercise-base falls prevention interventions were also reported by Stanghelle et al., [60] however, in their study the participants were only followed for 3-months post discharge. also reported that participants were able to maintain increased walking speed six-months after discharge after a six-month multi-dimensional exercise program in older adults at risk of falls. These findings are significant as walking speed is associated with decreased risk of disability, indicative of functional dependence and the ability to complete activities of daily living in community-dwelling older adults [60–62].

The current findings also highlight the importance of addressing fear of falling in any falls prevention program [4, 63]. Brown et al., [58] reported that participants with higher self-efficacy or confidence were more likely to adhere to ongoing exercise; a finding corroborated by other studies that have evaluated post-intervention follow-up in older people and those with neurological conditions [50, 64, 65]. Despite this, numerous other studies have reported contradictory outcomes [25, 66, 67]. The lack of consensus in the literature could be a result of the varied exercise-types, durations and intensities of exercise delivery, and the different follow-up periods, which varied in duration from eight weeks to 12 months [65]. These inconsistent findings in studies where participants’ ability to maintain benefits once interventions had ceased highlight the need for further research. In addition to rigorously controlled intervention trials, investigation of real-world clinical programs is needed, including input from key multidisciplinary clinicians, to better understand the effectiveness and safety of falls prevention programs for patients [68].

This evaluation study has limitations that may have affected the findings. Due to the nature of this study, which was an evaluation of existing clinical programs, there was an inherent sampling and convenience bias as all participants were recruited from the same demographic catchment area and hospital. Recruitment was not continuous across the recruitment period due to staffing availability issues. Recruitment took place in block periods or when staff were available to assess/contact potential participants. There was a high attrition rate due to a combination of medical complications and self-discharge. This resulted in the need for ongoing recruitment to achieve the target sample size, with the Ai Chi group only retaining eight participants at six-month follow-up. This study’s attrition rate (43%) was higher than expected, with a Cochrane systematic review reporting the average attrition rate in similar falls prevention interventions to be 10.9% [69]. The difference in this study could be attributed to the recruitment process. Typically, studies investigating falls in this population group are randomized-controlled trials, which recruit volunteers prior to intervention allocation [56, 70], whereas the participants of this study were already assessed and receiving the intervention regardless of their decision to participate in the study. Although, the non-randomized design of the current study may be considered a limitation, due to the high potential for selection or allocation bias, the aim of this study was to evaluate the real-world application of falls reduction programs, and therefore, the design could also be considered a strength as it represents clinical best fit for participants in falls prevention programs.

Through the evaluation of existing exercise-based falls prevention programs for community-dwelling at risk adults, this study demonstrates how recommendations from clinical trials can be implemented and translated into clinical practice. This study offers an insight into the real-world application and practice of falls reduction programs and outcomes. Despite recommendations advocating for increased service provision this study suggests that ten-weeks of either land- or water-based falls prevention programs, delivered in a clinical setting, can elicit an immediate and lasting improvement in physical outcomes associated with falls risk in older adults and adults with neurological conditions.

## Supporting information

S1 Checklist (DOC)

S1 Dataset (XLSX)

S1 File (DOCX)
