# Supplementary material for: The immediate and lasting balance outcomes of clinical falls-prevention programs: A non-randomised study
Source: PLoS One. 2024 Mar 14;19(3):e0299146. doi: 10.1371/journal.pone.0299146 (PMC10939286; doi:10.1371/journal.pone.0299146)
Supplement: S1 File — (DOCX) [file pone.0299146.s003.docx]

**Oberholster et al. 2023 - The immediate and lasting balance outcomes of clinical falls-prevention programs: A non-randomised study.**

Supplementary Table 1: Model fixed effects regression parameters with time and group effects

|  | 6-Minute Walk Test  [m] | |  | 6-Meter Walk - time  (% change) | |  | 6-Meter Walk - steps  (% change) | |  | Timed-up and Go  (% change) | |  | Five times Sit-To-Stand  (s) | |
| --- | --- | --- | --- | --- | --- | --- | --- | --- | --- | --- | --- | --- | --- | --- |
| **Fixed Effects** | Mean (95%CI) | SMD_rm_ |  | Mean (95%CI) | SMD_rm_ |  | Mean (95%CI) | SMD_rm_ |  | Mean (95%CI) | SMD_rm_ |  | Mean (95%CI) | SMD_rm_ |
| Intercept | 318.49 ^#^ (290.1, 346.88) |  |  | 5.93^#^ (5.47, 6.36) |  |  | 11.02 ^#^ (10.38, 11.59) |  |  | 12.30 ^#^ (11.36, 13.33) |  |  | 15.44 ^#^ (14.23, 16.65) |  |
| LLB^a^ | -91.96 ^ (-158.42, -25.49) | -2.48 |  | 33.64 ^^^ (11.63, 58.41) | 1.81 |  | 23.37^**^ (7.25, 41.91) | 1.62 |  | 53.73 ^#^ (28.4, 85.89) | 2.26 |  | 3.56^*^ (0.65, 6.46) | 1.05 |
| Ai Chi^a^ | -66.35 (-135.47, 2.77) | -1.79 |  | 33.64 ^^^ (10.52, 60) | 1.81 |  | 17.35^*^ (2.02, 36.34) | 1.23 |  | 34.99 ^^^ (11.63, 63.23) | 1.58 |  | 5.14 ^^^ (2.14, 8.14) | 1.51 |
| Post^b^ | 8.81 (-10.71, 28.32) | 0.24 |  | -6.76 (-13.93, 1.01) | -0.44 |  | -3.92 (-9.52, 3.05) | -0.31 |  | -10.42^*^ (-18.94, -1) | -0.58 |  | -1.79^*^ (-3.46, -0.13) | -0.53 |
| Follow-up^b^ | 19.51 (-1.6, 40.63) | 0.53 |  | -9.52^*^ (-17.3, -1) | -0.63 |  | -4.88 (-11.31, 2.02) | -0.38 |  | -11.31^*^ (-20.55, -1) | -0.63 |  | -1.63 (-3.44, 0.17) | -0.48 |
| IPAQ | 0 (0, 0.01) | 0.00 |  | 0.00 (0, 0) | 0.00 |  | 0.00 (0, 0) | 0.00 |  | 0.00 (0, 0) | 0.00 |  | 0 (0, 0) | 0.00 |
| MFES | 153.45 ^#^ (70.66, 236.25) | 4.14 |  | -18.13 (-41.14, 13.88) | -1.25 |  | -18.94 (-36.87, 5.13) | -1.62 |  | -22.12 (-46.21, 13.88) | -1.32 |  | -7.44^*^ (-13.72, -1.15) | -2.19 |
| LLB^a^ x Post^b^ | -45.89 | -1.24 |  | 9.42 (-8.61, 32.31) | 0.56 |  | 5.13 (-8.61, 22.14) | 0.38 |  | 11.63 (-11.31, 39.1) | 0.58 |  | 0.79 (-3.11, 4.68) | 0.23 |
| Ai Chi^a^ x Post^b^ | 15.09 (-30.52, 60.7) | 0.41 |  | 1.01 (-16.47, 23.37) | 0.06 |  | -3.92 (-17.3, 11.63) | -0.31 |  | -1.00 (-21.34, 25.86) | -0.05 |  | -0.52 (-4.58, 3.53) | -0.15 |
| LLB^a^ x Follow up^b^ | -22.84 (-70.27, 24.59) | -0.62 |  | -8.61 (-25.17, 12.75) | -0.56 |  | 4.08 (-11.31, 22.14) | 0.31 |  | 3.05 (-18.94, 32.31) | 0.16 |  | -0.86 (-5.03, 3.31) | -0.25 |
| Ai Chi^a^ x Follow up^b^ | 15.37 (-34.58, 65.32) | 0.41 |  | -1.98 (-21.34, 20.92) | -0.13 |  | -5.82 (-20.55, 11.63) | -0.46 |  | -10.42 | -0.58 |  | -0.77 (-5.18, 3.64) | -0.23 |
| **Random Effects** |  |  |  |  |  |  |  |  |  |  |  |  |  |  |
| Between participant SD | 90.57 |  |  | 1.25 |  |  | 1.19 |  |  | 1.23 |  |  | 8.60 |  |
| Within participant SD | 37.11 |  |  | 1.17 |  |  | 1.13 |  |  | 1.21 |  |  | 10.65 |  |
| ICC | 0.86 |  |  | 0.65 |  |  | 0.65 |  |  | 0.53 |  |  | 0.39 |  |
|  |  |  |  |  |  |  |  |  |  |  |  |  |  |  |

LLB=low level balance, Post= , IPAQ= International Physical Activity Questionnaire , MFES= Modified Falls Efficacy Questionnaire , SD= standard deviation, CI= 95% confidence interval, SMD_rm_= standardised mean difference (repeated measures), ^a^ reference = High Level Balance, ^b^ reference = Pre-intervention, # p <.001 , ^ p<.01 , *p<0.5

Supplementary Table 2. Mean scores for groups at each assessment point

|  | | High Level Balance | | | Low Level Balance | | | Ai Chi | |
| --- | --- | --- | --- | --- | --- | --- | --- | --- | --- |
| Measure | Post-intervention  (n=10) | | 6-month follow-up  (n=8) | Post-intervention  (n=10) | | 6-month follow-up  (n=8) | Post-intervention  (n=10) | | 6-month follow-up  (n=8) |
| 6-min walk (m)* | 415 (345-487) | | 434.0 (366.5-501.5) | 246 (183-310) | | 279.6 (209.3-349.9) | 339 (269-409) | | 334.4 (247.9-420.9) |
| 6-meter walk (s)* | 4.6 (4.1-5.2) | | 4.7 (4.2-5.1) | 7.3 (5.9-8.6) | | 5.7 (3.7-7.6) | 6.1 (5.0-7.1) | | 5.9 (5.0-6.9) |
| 6-meter walk (steps)** | 9.0 (8.25-10.0) | | 10.0 (8.0-10.0) | 12.5 (11.0-14.2.0) | | 12.0 (9.5-13.0) | 10.5 (8.5-12.7) | | 10.5 (8.25 - 12.5) |
| Timed-up and go (s)* | 8.7 (7.6-9.8) | | 9.3 (7.6-10.9) | 16.9 (12.9-21.0) | | 14.1 (9.7-18.5) | 12.2 (10.3-14.0) | | 11.29 (9.3-13.3) |
| 5 x sit to stand (s)* | 11.2 (9.3-13.2) ᵩ | | 12.3 (10.3-14.2) | 16.7 (14.8-18.6) | | 15.7 (12.7-18.7) | 15.9 (12.7-19.1) | | 16.9 (9.9-23.8) |
| MFES (%) * | 83.4 (75.4-91.5) | | 85.4 (72.9-97.9) | 79.2 (68.7-89.7) | | 80.9 (71.7-90.1) | 83.2 (76.3-90.1) | | 79.8 (69.6-89.9) |
| IPAQ (MET/min/week) * | 3522.9 (1631.3-5414.5) | | 2272.5 (473.8 -4071.1) | 1727.1 (826.5-2627.7) | | 1349.3(492.5-2206.1) | 2596.0 (1830.8-3361.3) | | 2443.6 (983.7-3903.5) |

* Reported in mean and 95% CI, **Reported in median and IQR, MFES = Modified Falls Efficacy Scale, IPAQ = International Physical Activity Scale
